# Optical coherence tomography angiography findings of choroidal neovascularization in pseudoxanthoma elasticum

**DOI:** 10.1186/s40942-015-0011-x

**Published:** 2015-08-07

**Authors:** Orly Gal-Or, Chandrakumar Balaratnasingam, K Bailey Freund

**Affiliations:** 1 LuEsther T. Mertz Retinal Research Center, Manhattan Eye, Ear and Throat Hospital, New York, NY USA; 2 Vitreous-Retina-Macula Consultants of New York, 460 Park Avenue, 5th Floor, New York, NY 10022 USA; 3 Centre for Ophthalmology and Visual Sciences, Lions Eye Institute, University of Western Australia, Perth, Australia; 4 Department of Ophthalmology, New York University School of Medicine, New York, NY USA; 5 Ophthalmology Department, Rabin Medical Center, Petach Tikva, Israel

**Keywords:** Angioid streaks, Bruch’s membrane, Choroidal neovasculariztion, Multimodal imaging, Optical coherence tomography angiography

## Abstract

Angioid streaks (AS) are the most common ocular manifestation in patients with pseudoxanthoma elasticum (PXE). The major cause of severe visual loss in patients with AS is choroidal neovascularization (CNV). We report the optical coherence tomography angiography (OCTA) findings of CNV in a patient with PXE and angioid streaks. A 51-year-old man with PXE presented with visual disturbance in his right eye. Best corrected visual acuity was 20/30 OD and 20/30 OS. Funduscopic examination revealed angioid streaks and type 1 NV in his right eye. Multimodal imaging including OCTA demonstrated CNV nasal to the fovea. The morphology and configuration of CNV followed the path of the AS. OCTA combined with other multimodal imaging modalities may be a useful tool for diagnosing CNV secondary to angioid streaks in patients with pseudoxanthoma elasticum. The configuration of CNV in these may follow the path of angioid streaks implicating Bruch’s membrane disruption as an important anatomical change in the pathogenesis of CNV.

## Background

Pseudoxanthoma elasticum (PXE) is a rare inherited multisystem disorder. There are many ocular manifestations associated with PXE of which angioid streaks (AS) are the most common. AS represent breaks within calcified and thickened elastic fibers of Bruch’s membrane. They often do not affect visual function, but they may be complicated by the development of choroidal neovascularization (CNV), which is a major cause of visual loss in these patients. Optical coherence tomography angiography (OCTA) is relatively new non-invasive imaging modality that demonstrates flow-characteristics of the vascular network. In this report we demonstrate the morphology and spatial relationships of CNV and angioid streaks using OCTA.

## Case presentation

Pseudoxanthoma elasticum is an inherited multisystem disorder with a prevalence range between 1:25,000 and 1:100,000 [1]. There are many ocular manifestations associated with PXE of which angioid streaks (AS) are the most common. Angioid streaks are identified in approximately 85% of eyes with PXE and represent breaks within calcified and thickened elastic fibers of Bruch’s membrane [2, 3]. AS often do not affect visual function, even if they cross into the foveal area, but they may be complicated by the development of choroidal neovascularization (CNV) [4]. The occurrence of CNV is a major cause of visual loss in PXE and our knowledge concerning the pathogenesis, morphology and natural course of this complication remains limited [5].

Optical coherence tomography angiography (OCTA) is relatively new imaging modality that exploits flow-characteristics within the regional circulation to construct non-invasive images of the vascular network [6]. *En face* images generated by OCTA also allows us to study the spatial relationships between vasculature and adjacent retinal/choroidal layers with greater precision than dye angiography. In this report, we integrate OCTA with other multimodal imaging findings to improve our understanding of CNV in the setting of angioid streaks.

**Fig. 1:**
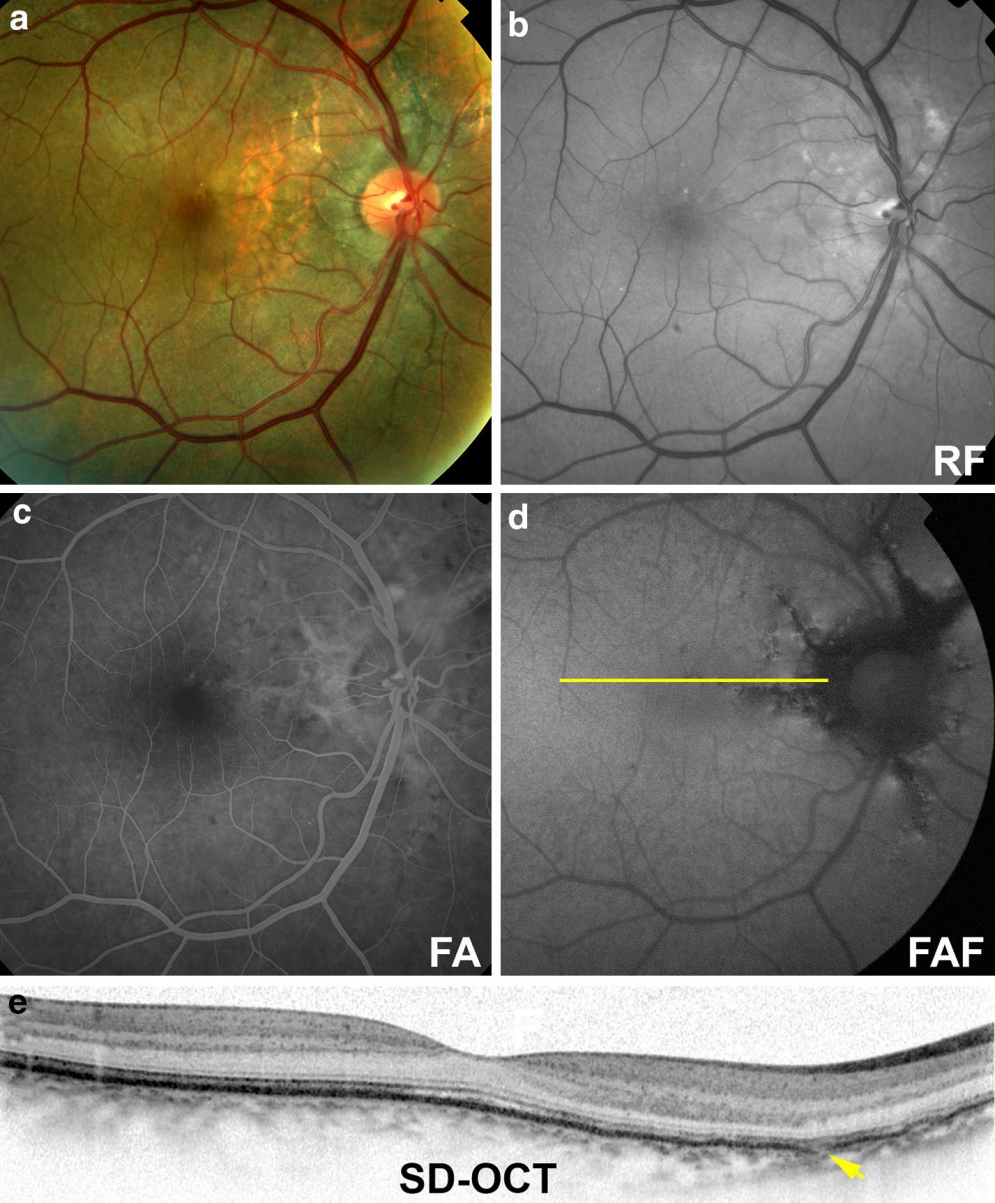
Multimodal imaging findings of angioid streaks at baseline visit. Color (**a**) and red-free (RF) photographs (**b**) of the right eye show angioid streaks at the peripapillary region radiating into the fovea. Late-phase fluorescein angiogram (**c**; FA) shows variable staining of the angioid streaks with no leakage. Fundus autofluorescence (**d**; FAF) imaging shows areas of hypoautofluorescence in the peripapillary area that are greater in size than the corresponding AS seen on color photography. AS correlate to breaks within the Bruch’s membrane (*yellow arrow*) as seen on spectral domain optical coherence tomography (**e**; SD-OCT). The yellow line indicates the location of the SD-OCT scan.

The study was approved by the Western Institutional Review Board and written informed consent was provided by the patient. A 51-year-old male patient had received care for the ocular complications of pseudoxanthoma elasticum for 9 years. Best corrected visual acuity was 20/30 OD and 20/30 OS. On presentation, his right eye demonstrated AS on multimodal imaging (Fig. 1). Color fundus and red-free photographs revealed the AS in the peripapillary area radiating to the periphery of the posterior pole and towards the fovea with slight foveal mottling. Fluorescein angiography showed variable staining of the AS without leakage in any phase. Fundus autofluorescence showed distinct areas of hypoautofluorescence corresponding to the AS with focal spots of increased autofluorescence alongside the AS. SD-OCT showed breaks in Bruch’s membrane (BrM) with preservation of the overlying RPE. His left eye showed subfoveal CNV that was treated with photodynamic therapy (PDT) and several anti-vascular endothelial growth factor (anti-VEGF) injections with eventual subretinal fibrosis. Final best corrected visual acuity in the left eye was 20/30.

Five years later, the patient presented with visual disturbances involving his right eye. Dilated funduscopic examination revealed juxtafoveal CNV. The patient underwent monthly injections of bevacizumab (1.25 mg/0.05 ml) for 11 months that achieved regression of the lesion.

**Fig. 2:**
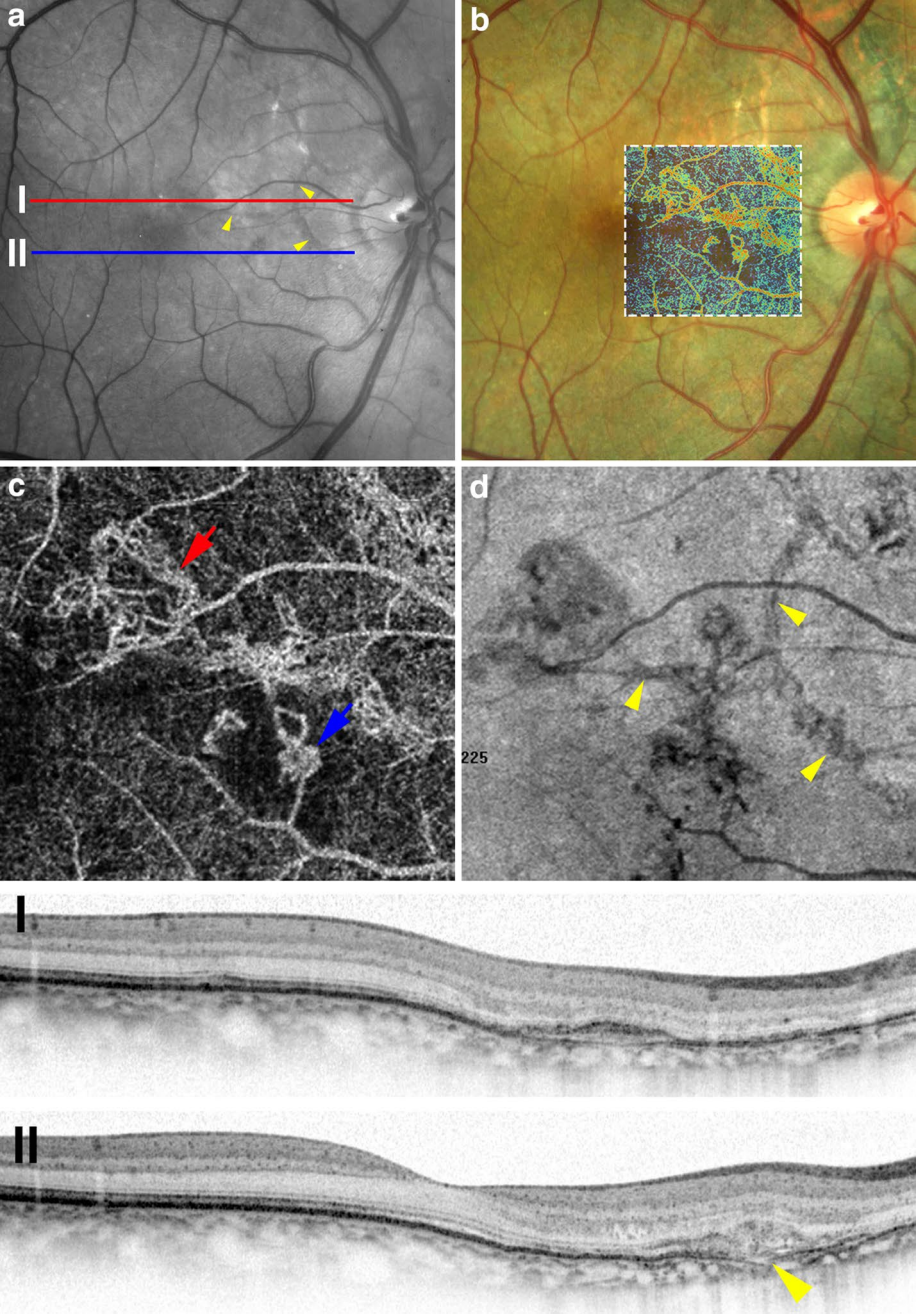
Multimodal imaging findings of choroidal neovascularization. Angioid streaks are observed on the red-free image (**a**); however, no hemorrhage is evident. Overlay of false-colored optical coherence tomography angiography (OCTA) over the color photograph (**b**) outlines the area of choroidal neovascularization (CNV). *En Face* OCTA (**c**) demonstrates two area of CNV (*red* and *blue arrows*) that demonstrate a tangled morphology of vascular networks. Sites of CNV closely correlate to sites of angioid streaks (**d**; *arrowheads*) when OCTA is compared to *en face* reflectance images. Structural OCT scans (I and II) confirm a mixed type 1 (I) and type 2 (II) neovascular lesion which arise in proximity to sites of Bruch’s membrane disruption.

**Fig. 3:**
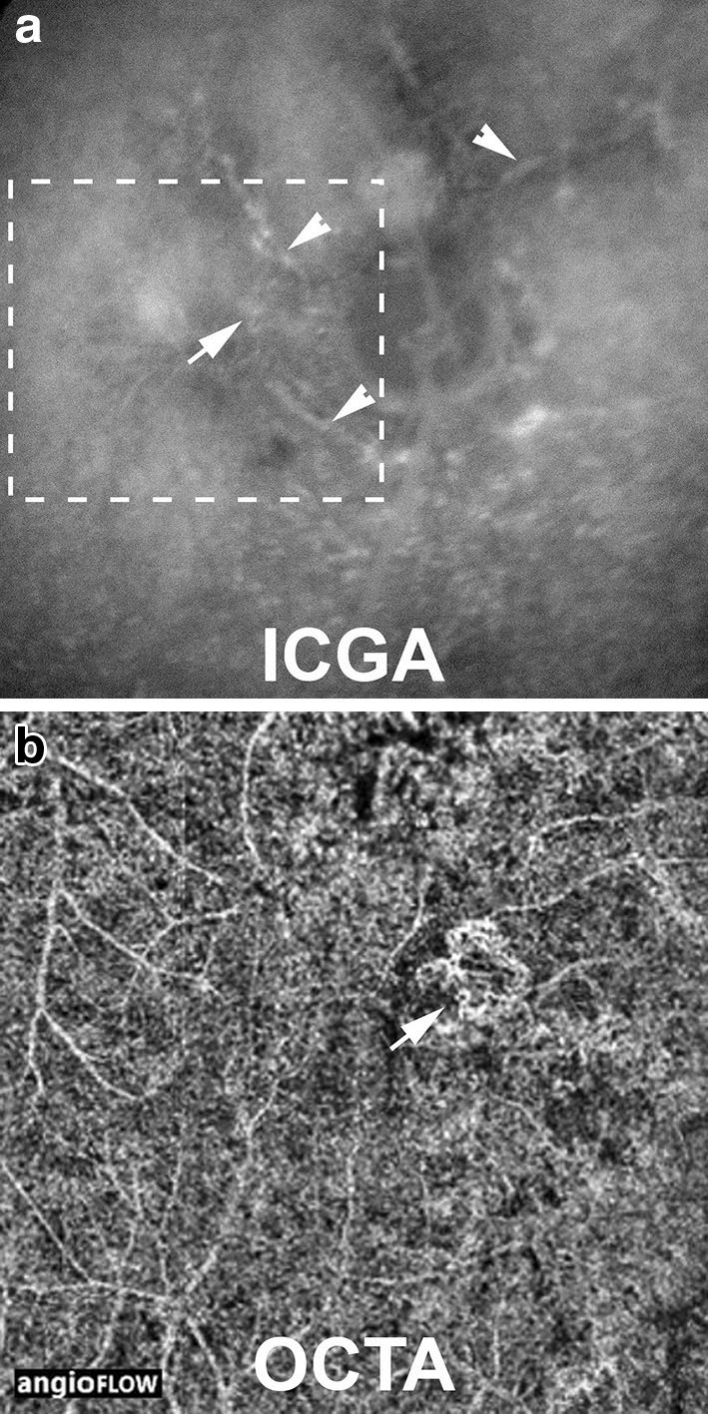
Comparison between indocyanine green angiography and optical coherence tomographic angiography for visualizing type 1 neovascularization. Areas of type 1 NV (*arrow*) may be difficult to discern on ICGA (**a**) due to hyperfluorescent staining of angioid streaks (*arrowheads*) within areas of NV. In contrast, type 1 NV demonstrates a characteristic tangled morphology appearance on OCTA (**b**) that is easily appreciated. Inset on ICGA corresponds to the region of interest on OCTA image. Note the projection artifact of retinal vessels on the OCTA image.

Two years following this, there was recurrence of CNV in the same eye. Multimodal imaging characteristics of this CNV are provided in Figs. 2 and 3. There was no evolution in the size of the angioid streaks since presentation. OCTA demonstrated CNV nasal to the fovea. The morphology of CNV was characterized by a tangled network of vessels [7]. The configuration of CNV closely followed the trajectory of AS. Structural OCT revealed type 1(between RPE and BrM) and type 2 (sub-retinal) neovascularization. Breaks in BrM were seen on the outer aspect of the area of neovascularization. Visualization of the ultrastructural features of CNV using indocyanine green angiography was limited in this case due to the hyperfluorescence of angioid streaks at the site of CNV.

The patient received monthly intravitreal ranibizumab (0.5 mg/0.05 ml) injections on a *pro re nata* protocol for 2 months. The CNV was quiescent for 5 months. On his last visit date his best corrected visual acuity was 20/25 OD 20/30 OS.

Choroidal neovascularization (CNV) is an important cause of vision loss in eyes with angioid streaks and occurs in approximately 42–84% of cases. While type 2 NV is the most common presenting anatomical subtype of NV to occur in the setting of angioid streaks, type 1 and mixed (type 1 and type 2) lesions occur in up to 16% of eyes [8]. Bruch’s membrane (BrM) plays a critical role in retinal and choroidal physiology and regulates nutrient exchange and metabolic homeostasis [9]. BrM also serves an important mechanical function and separates the choroidal circulation from the outer retina. Interruptions in the mechanical integrity of BrM can result in dysregulation of regional growth factor gradients that together with a potential communication between retinal and choroidal compartments can culminate in CNV. Focal defects in BrM have been implicated in the pathophysiology of CNV in a range of conditions; however, this relationship is best exemplified in conditions with angioid streaks. The spatial relationship between Bruch’s membrane and CNV can be precisely studied using OCTA where, as shown in this report, there is a close relationship between the morphology of BrM defects and the topographic pattern of CNV. The morphology of type 1 NV, with respect to the tangled morphology of pathological vessels, [10] shows many similarities to what has been described in AMD. This suggests that there is some overlap in biochemical stimuli that promote pathological angiogenesis in AMD and conditions associated with angioid streaks.

## Conclusions

The multimodal imaging characteristics of angioid streaks have been previously reported [8, 11–13]. To our knowledge, this is the first report to demonstrate the morphology and spatial relationships of CNV and angioid streaks using OCTA. Our observations support the evidence that breaks in BrM are indeed the underlying pathology of AS [11]. The findings in this report implicate a critical role served by Bruch’s membrane defects in the pathophysiology of CNV. In conclusion, multimodal imaging in combination with OCTA provides us with an excellent tool for the early detection and monitoring of AS related CNV. Those tools may enable improved visual outcomes for the management of CNV secondary to AS that often occurs in relatively young patient population with PXE.

## Consent

Written informed consent was obtained from the patient for publication of this Case report and any accompanying images. A copy of the written consent is available for review by the Editor-in-Chief of this journal.

## Abbreviations

AS: angioid streaks
PXE: pseudoxanthoma elasticum
CNV: choroidal neovascularization
OCTA: optical coherence tomography angiography
BrM: Bruch’s membrane
OD: oculus dexter (right eye)
OS: oculus sinister (left eye)
SD-OCT: spectral domain optical coherence tomography
RPE: retinal pigment epithelium
PDT: photodynamic therapy
Anti-VEGF: anti-vascular endothelial growth factor
